# MicroRNA-139-5p Alleviates High Glucose-Triggered Human Retinal Pigment Epithelial Cell Injury by Targeting LIM-Only Factor 4

**DOI:** 10.1155/2021/1629783

**Published:** 2021-10-23

**Authors:** Kan Shao, Gong Chen, Lili Xia, Cheng Chen, Shan Huang

**Affiliations:** ^1^Department of Endocrinology, Shanghai Tongren Hospital, Shanghai Jiao Tong University School of Medicine, Shanghai, China; ^2^School of Environmental and Materials Engineering, College of Engineering, Shanghai Polytechnic University, Shanghai, China

## Abstract

Diabetic retinopathy (DR) is a type of diabetes complication, which can result in loss of vision in adults worldwide. Increasing evidence has revealed that microRNAs (miRs) can regulate DR progression. Thus, the present study was aimed at assessing the possible mechanism of miR-139-5p in high glucose- (HG-) incubated retinal pigment epithelial (ARPE-19) cells. The present results demonstrated that miR-139-5p expression was notably reduced in the serum samples of patients with DR, as well as in ARPE-19 cells treated with HG in a time-dependent manner. Moreover, miR-139-5p was markedly overexpressed by transfection of miR-139-5p mimics into ARPE-19 cells. Overexpression of miR-139-5p markedly induced cell viability and repressed HG-triggered apoptosis. Furthermore, overexpression of miR-139-5p relived HG-enhanced oxidative stress injury. It was found that HG induced malondialdehyde levels but decreased superoxide dismutase and glutathione peroxidase activities in ARPE-19 cells. In addition, overexpression of miR-139-5p could markedly decrease intracellular stress. The results demonstrated that overexpression of miR-139-5p effectively repressed HG-activated inflammation, as indicated by the upregulation of inflammation cytokines, including TNF-*α*, IL-6, and Cox-2, in ARPE-19 cells. Subsequently, it was identified that LIM-only factor 4 (LMO4) could act as a downstream target for miR-139-5p. LMO4 expression was significantly increased in patients with DR and HG-treated ARPE-19 cells. Mechanistically, knockdown of LMO4 reversed the biological role of miR-139-5p in proliferation, apoptosis, oxidative stress, and release of inflammation factors *in vitro*. Collectively, these results suggested that miR-139-5p significantly decreased ARPE-19 cell injury caused by HG by inducing proliferation and suppressing cell apoptosis, oxidant stress, and inflammation by modulating LMO4.

## 1. Introduction

Diabetic retinopathy (DR), a common complication resulting from diabetes, is a leading cause of blindness worldwide [1]. As well established, loss of pericytes, death of endothelial cells, cellular capillary formation, basement membrane thickness, and retinal neovascularization are pathologies of DR [2]. At present, the current therapeutic approaches for DR treatment include anti-VEGF therapy and laser photocoagulation. However, significant adverse effects are observed among these [3]. Therefore, it is important for the treatment of DR to identify its underlying molecular mechanisms.

Among different cellular components of human eyes, retinal pigment epithelial (RPE) cells are demonstrated to be most vulnerable to hyperglycemia or HG conditions [4, 5]. Meanwhile, several signaling pathways are reported to participate in the process of HG-induced RPE injuries, including the production of reactive oxygen species and the dysfunction of metabolic processing [6]. However, the full genetic/molecular network of hyperglycemia-associated injures in RPE has yet to be elucidated.

MicroRNAs (miRNAs/miRs) can target genes by binding with the 3′-untranslated regions (UTRs) of their targeting mRNAs [7, 8]. Recently, it has been reported that miRNAs serve important roles in multiple biological processes, such as proliferation, apoptosis, and cell development [9–11]. In addition, miRNAs can exert important roles in various diseases, such as cancer, diabetes, and complications of diabetes [12–14]. In recent years, it has revealed that numerous miRNAs participate in the pathogenesis of DR [15, 16]. For example, miR-21 exhibits a pathogenic role in DR progression by downregulating peroxisome proliferator-activated receptor *α* [17], while miR-126 represses cell progression in a DR model by negatively regulating insulin receptor substrate 1 (IRS-1) [18]. In addition, miR-384-3p decreases retinal neovascularization by regulating hexokinase 2 [19].

The key roles of miRNAs are also being investigated with regard to vascular development. The present data demonstrated the effect of miR-139-5p in DR progression. miR-139-5p has been described in a number of studies examining tumors [20, 21], and previous studies have revealed that miR-139-5p is involved in numerous diseases. For instance, miR-139-5p can repress oral squamous carcinoma cell tumorigenesis and development by targeting homeobox A9 [22]. miR-139-5p can also repress liver cancer aerobic glycolysis and cell progression by interacting with ETS protooncogene 1, transcription factor [23]. Additionally, miR-139-5p can suppress airway smooth muscle cell proliferation and increase cell apoptosis by targeting Brg1 [24]. miR-139-5p is also able to coordinate apelin receptor/C-X-C motif chemokine receptor 4 crosstalk during vascular maturation [25]. In hemangioma stem cells, miR-139-5p can affect cell proliferation, migration, and adipogenesis through targeting insulin-like growth factor 1 receptor (IGF-1R) [26]. Few reports have reported the relationship between miR-139-5p and human retinal pigment epithelial cells. The relationship between miR-139-5p and human retinal microvascular endothelial cells has been reported. The miRNA profiles of human retinal microvascular endothelial cells (HRMVECs) under angiogenic conditions exhibit an increase in miR-139-5p [27]. However, its value in DR progression is yet to be widely investigated.

The present study investigated the underlying mechanism of miR-139-5p in DR progression, with a focus on the relationship between miR-139-5p and human retinal pigment epithelial cells. Firstly, the expression of miR-139-5p in patients with DR and ARPE-19 cells treated with HG was determined. Then, the biological roles of miR-139-5p in HG-induced cell damage were studied. The present data may provide novel insights for effective strategies to treat DR.

## 2. Materials and Methods

### 2.1. Clinical Serum Samples

In total, 30 patients with DR and 30 healthy individuals were enrolled in the present research. Between these two groups, there were no significant differences in age and sex. The present study was approved by the Medical Ethics Committee of Shanghai Tongren Hospital, Shanghai Jiao Tong University School of Medicine. The samples were obtained from Shanghai Tongren Hospital, Shanghai Jiao Tong University School of Medicine, from September 2012 to September 2018. All patients were diagnosed based on the standard established by the Chinese Medical Association (2014). Inclusion criteria were as follows: (1) patients with diabetic retinopathy or diabetes only diagnosed in the Shanghai Tongren Hospital, Shanghai Jiao Tong University School of Medicine, and (2) patients who understood the experimental protocol and were willing to participate. Exclusion criteria were as follows: (1) patients who were treated within 90 days before admission and (2) patients having other diseases. Healthy volunteers served as a control group, and they exhibited all physiological parameters within a normal range after systemic physiological examinations. Patients with other more severe diseases were excluded. Written consent was obtained from the participants. The characteristics of clinical volunteers including age and sex distribution are presented in Table 1.

### 2.2. Cell Culture

ARPE19 cells were purchased from the Type Culture Collection of Chinese Academy of Sciences. Endothelial cell medium (Sigma-Aldrich; Merck KGaA) was used for cell culture, which was supplemented with 10% FBS (Sigma-Aldrich; Merck KGaA), 100 U/ml penicillin, and 100 mg/ml streptomycin. A humified atmosphere at 37°C with 5% CO_2_ was utilized to maintain the cells.

### 2.3. Cell Transfection

Mimics and inhibitors of miR-139-5p, as well as their corresponding negative controls (NCs), were obtained from Shanghai GenePharma Co., Ltd. Cells were transiently transfected with *miR-139-5p* mimics or inhibitors at a final concentration of 100 nM. The sequences were as follows:

miR-139-5p mimic sense: UCUACAGUGCACGUGUCUCCAGU

miR-139-5p mimic antisense: ACUGGAGACACGUGCACUGUAGA

miR-139-5p inhibitor: ACUGGAGACACGUGCACUGUAGA

miR-139-5p mimic negative control sense: UUUGUACUACACAAAAGUACUG

miR-139-5p mimic negative control antisense: CAGUACUUUUGUGUAGUACAAA

miR-139-5p inhibitor negative control: CAGUACUUUUGUGUAGUACAA.

LIM-only factor 4 (LMO4) small interfering (si) RNA was synthesized by Shanghai GenePharma Co., Ltd. The sequences of LMO4 siRNA were as follows:

5′-GUCGAUUCCUGCGAGUGAAdTdT-3′

siRNA-control: 5′-GCATCAACAACCGAACATT-3′.

Cells were transfected with LMO4 siRNA at a final concentration of 50 nM. Cells were seeded in 6-well plates at a density of 1 × 10^6^ cells/ml per well, and the cell density reached 60-70% confluence next day in the incubator. Then, transfection was performed using Lipofectamine® 3000 (Invitrogen; Thermo Fisher Scientific, Inc.) at r5oom temperature. Further experiments were carried out 48 h following transfection.

### 2.4. Cell Counting Kit (CCK)-8 Assay

Cell survival was examined using a CCK-8 assay (Dojindo Molecular Technologies, Inc.) according to the manufacturer's instructions. Briefly, cells were grown in the 96-well plate. Then, 10 *μ*l CCK-8 reagent was supplemented into the wells for 2 h under a standard culture condition. In order to evaluate cell viability, the optical density values were assessed at 450 nm.

### 2.5. Apoptosis Analysis

Cell apoptosis was assessed using an Annexin-V/PI Apoptosis Detection kit (Nanjing KeyGen Biotech Co., Ltd.). In brief, cells were collected after being transfected for 48 h and suspended in 1X binding buffer. Then, Annexin-V and PI staining was conducted. Cells were incubated for 15 min in the dark. A FACSCalibur flow cytometer (BD Biosciences) was used for the analysis of cell apoptosis.

### 2.6. Determination of ROS, Malondialdehyde (MDA), Superoxide Dismutase (SOD), and Glutathione Peroxidase (GSH-Px)

After cells were washed using PBS twice, cells were suspended using DCFHDA (10 mM) solution and incubated at 37°C for 20 minutes. An ROS Assay Kit (Beyotime, Shanghai, China) was carried out. A MDA assay kit (Beyotime Institute of Biotechnology) was utilized to test MDA contents, while SOD activity was examined using a SOD assay kit (Nanjing Jiancheng Bioengineering Institute). Moreover, GSH-PX was measured using a GSH-PX assay kit (Nanjing Jiancheng Bioengineering Institute).

### 2.7. ELISA

TNF-*α*, IL-6, and Cox-2 levels were detected using the corresponding ELISA kits (Abcam) according to the manufacturers' protocol.

### 2.8. Luciferase Reporter Gene System

The wild-type (WT) or mutant (MUT) 3′-UTR of LMO4 was cloned into a pGL3 vector (Promega Corporation). Then, in order to conduct the luciferase reporter assay, the luciferase reporter vectors and miR-139-5p mimics or NC were cotransfected into the cells. After 48 h, a dual-luciferase reporter assay system (Promega Corporation) was used to assess the luciferase activity.

### 2.9. Reverse Transcription-Quantitative PCR (RT-qPCR)

TRIzol® (Invitrogen; Thermo Fisher Scientific, Inc.) was used to obtain total RNA. Then, TaqMan Reverse Transcription reagents (Applied Biosystems; Thermo Fisher Scientific, Inc.) were used to reverse transcribe the total RNA into cDNA. In order to quantify the target cDNA, the SYBR Green PCR Master Mix (Applied Biosystems; Thermo Fisher Scientific, Inc.) was used on an Applied Biosystems 7900 Real-Time qPCR system (Applied Biosystems; Thermo Fisher Scientific, Inc.). The primer sequences are listed in Table 2. Relative gene expression level was analyzed using the 2^−ΔΔCq^ method.

### 2.10. Western Blotting

Whole-cell lysate was prepared using ice-cold RIPA buffer with protease inhibitors. Then, protein samples were resolved via 10% SDS-PAGE electrophoresis. Nitrocellulose membranes were employed to transfer the proteins. Next, membranes were incubated with the following primary antibodies: rabbit anti-LMO4 monoclonal antibody (Abcam, ab131030) and anti-GAPDH polyclonal antibody (Abcam, ab9485). Subsequently, incubation with goat anti-rabbit IgG (Abcam, ab205718) or goat anti-mice IgG (Abcam, ab205719) was conducted. Finally, immunoreactive bands were observed using an ECL reagent (Pierce; Thermo Fisher Scientific, Inc.).

### 2.11. Statistical Analysis

Significant differences were analyzed using unpaired Student's *t*-test between two groups. Moreover, one-way ANOVA was performed for multiple comparisons followed by Tukey's post hoc test. The *χ*^2^ test was used to analyze categorical variables. Statistical analysis was conducted using GraphPad Prism version 5.0 (GraphPad Software, Inc.) and SPSS 22.0 (IBM Corp.). *P* < 0.05 was considered to indicate a statistically significant difference.

## 3. Results

### 3.1. miR-139-5p Is Notably Decreased in DR

**Figure 1:**
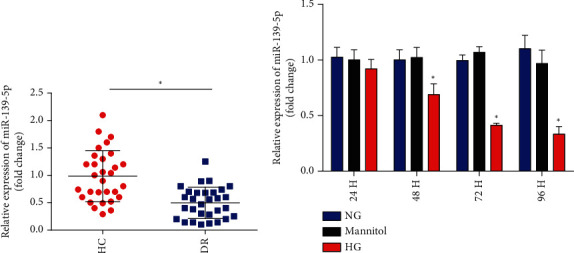
Identification of miR-139-5p in DR. (a) Analysis of miR-139-5p levels in serum from HC (*n* = 30) and patients with DR (*n* = 30). U6 was used as a loading control. (b) Expression levels of miR-139-5p in ARPE-19 cells. ARPE-19 cells were incubated with 25 mM glucose for 0, 24, 48, 72, and 96 h. A total of three independent experiments were conducted. Error bars represent the mean ± SD of ≥3 experiments. ^∗^*P* < 0.05. miR: microRNA; HC: healthy controls; DR: diabetic retinopathy.

The serum level of miR-139-5p was markedly decreased in patients with DR compared to the healthy controls (*n* = 30) (Figure 1(a)). Moreover, ARPE-19 cells were exposed to 5.5 mmol/l of glucose (normal control), 5.5 mmol/l of glucose, and 24.5 mmol/l of mannitol and 25 mmol/l of glucose (HG). The RT-qPCR results demonstrated that 25 mM HG treatment decreased miR-139-5p expression in ARPE-19 cells in a time-dependent manner (Figure 1(b)). These results suggested that miR-139-5p participated in DR.

### 3.2. Influence of miR-139-5p Mimics on ARPE-19 Cell Proliferation and Apoptosis

**Figure 2:**
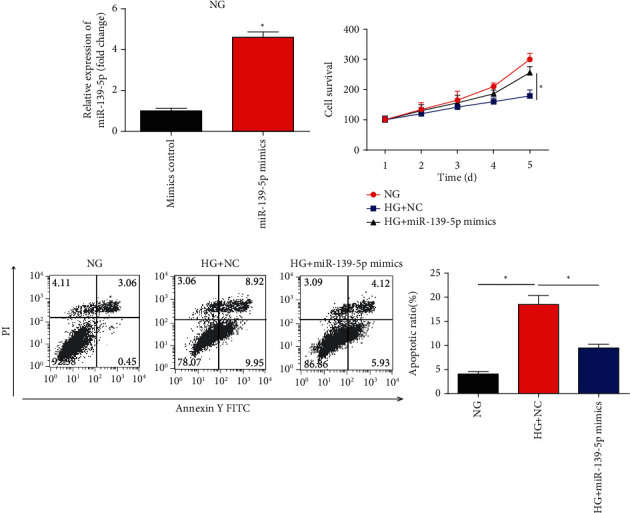
Impacts of miR-139-5p mimics on ARPE-19 cell proliferation and apoptosis. (a) Expression levels of miR-139-5p in ARPE-19 cells. Cells were transfected with miR-139-5p mimics under NG. (b) Cell viability was tested using a Cell Counting Kit-8 assay. (c) Cell apoptosis was assessed via flow cytometry. A total of three independent experiments were performed. Error bars represent the mean ± SD of ≥3 experiments. ^∗^*P* < 0.05 vs. control; ^#^*P* < 0.05 vs. HG+NC. HG: high glucose; NG: normal glucose; miR: microRNA.

To examine the effect of miR-139-5p on HG-induced cell injury, miR-139-5p was overexpressed using miR-139-5p mimics. As confirmed in Figure 2(a), the expression of miR-139-5p was significantly induced by the mimics in ARPE-19 cells under NG, which proved the transfection efficiency of miR-139-5p mimics. The CCK-8 results demonstrated that miR-139-5p markedly enhanced cell proliferation, which was decreased by HG incubation (Figure 2(b)). Flow cytometry was conducted to determine cell apoptosis. It was identified that HG notably increased cell apoptosis compared to NG treatment (Figure 2(c)). miR-139-5p mimics decreased the HG-induced apoptosis in ARPE-19 cells (Figure 2(c)). These findings indicated that miR-139-5p could increase cell proliferation and depress cell apoptosis under HG stimulation.

### 3.3. Effect of miR-139-5p Mimics on ARPE-19 Cell Oxidative Stress Stimulated by HG Treatment

**Figure 3:**
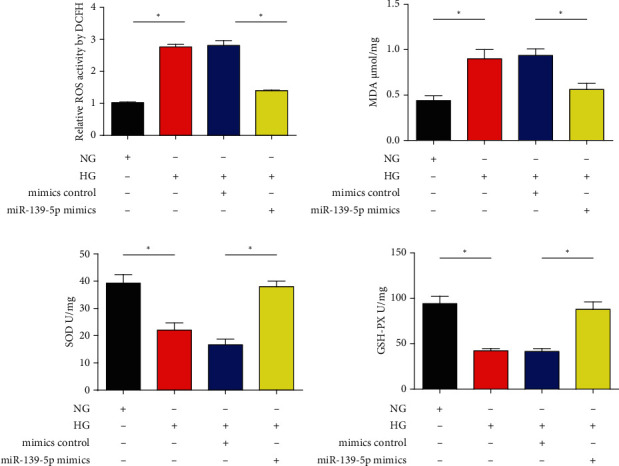
Effects of miR-139-5p mimics on HG-triggered oxidative stress. (a) ROS production. (b) MDA content. (c) Activities of SOD. (d) GSH-PX activity. A total of three independent experiments were carried out. Error bars represent the mean ± SD of ≥3 experiments. ^∗^*P* < 0.05. miR: microRNA; MDA: malondialdehyde; SOD: superoxide dismutase; GSH-PX: glutathione peroxidase.

Moreover, to identify the role of miR-139-5p in HG-stimulated oxidative stress, oxidative stress production was assessed *in vitro*. It was observed that HG raised ROS production and MDA levels, while miR-139-5p diminished the accumulation of them (Figures 3(a) and 3(b)). Then, the activities of SOD, which is an antioxidant enzyme, were determined. As presented in Figure 3(c), HG decreased the activities of SOD, which could be reversed by the mimics. Moreover, overexpression of miR-139-5p abrogated the effects of HG on GSH-PX activity (Figure 3(d)). These findings suggested that miR-139-5p had an antioxidant effect in ARPE-19 cells.

### 3.4. Effect of miR-139-5p Mimics on HG-Simulated Inflammation in the ARPE-19 Cell

**Figure 4:**
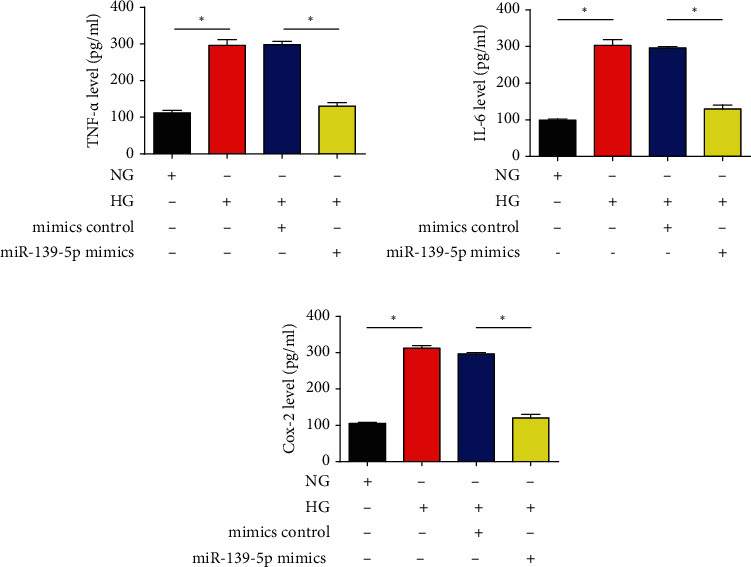
Effects of miR-139-5p mimics on HG-triggered inflammation. The levels of the proinflammatory cytokines (a) TNF-*α*, (b) IL-6, and (c) Cox-2 were measured using ELISA. A total of three independent experiments were carried out. Error bars represent the mean ± SD of ≥3 experiments. ^∗^*P* < 0.05. miR: microRNA; HG: high glucose.

Subsequently, the effect of miR-139-5p on the inflammatory process was examined by measuring inflammation cytokines. An ELISA was utilized to measure TNF-*α*, IL-6, and Cox-2 levels. It was found that HG could result in elevated protein levels of TNF-*α*, IL-6, and Cox-2, which were repressed by miR-139-5p (Figures 4(a)–4(c)). These results suggested that miR-139-5p exerted an anti-inflammation effect.

### 3.5. LMO4 Is a Target of miR-139-5p

**Figure 5:**
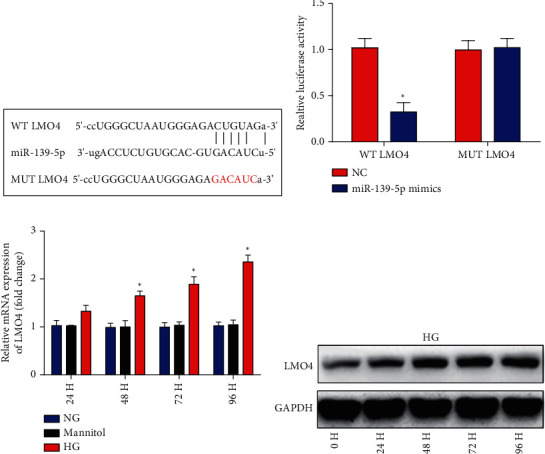
LMO4 is a direct target gene of miR-139-5p. (a) Schematic diagram showing miR-139-5p putative binding sites in LMO4 3′-UTR. (b) Interaction between the miR-139-5p and LMO4 3′-UTR was determined using a dual-luciferase reporter assay. (c) Reverse transcription-quantitative PCR was used to estimate LMO4 mRNA expression in ARPE-19 cells under NG or HG during various time points. (d) Western blotting was used to assess LMO4 protein expression in ARPE-19 cells treated with HG at different hours. A total of three independent experiments were carried out. Error bars represent the mean ± SD of ≥3 experiments. ^∗^*P* < 0.05. miR: microRNA; LMO4: LIM-only factor 4; UTR: untranslated region.

Next, bioinformatics analysis was used to predict the potential target of miR-139-5p. The binding site of miR-139-5p in the LMO4 3′-UTR is presented in Figure 5(a). It was identified that miR-139-5p significantly restrained the luciferase reporter activity of the WT LMO4 3′-UTR. However, no effect was indicated in the MUT reporter (Figure 5(b)). It was further demonstrated that LMO4 expression was enhanced by HG in ARPE-19 cells (Figures 5(c) and 5(d)). These findings suggested that miR-139-5p could directly bind with the 3′-UTR of LMO4.

### 3.6. LMO4 Is Involved in HG-Induced Injury

**Figure 6:**
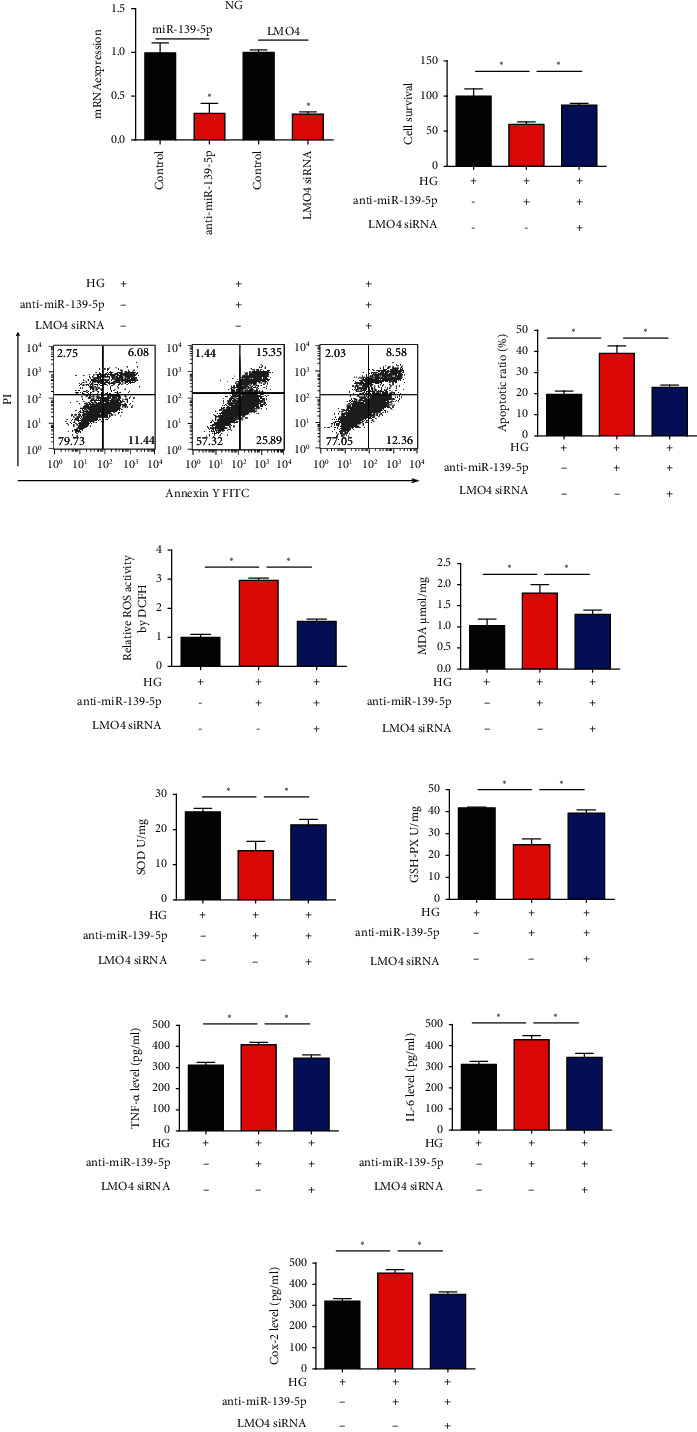
Loss of LMO4 reverses the role of miR-139-5p in HG-induced injury. (a) miR-139-5p and LMO4 mRNA expression in ARPE-19 cells. Cells were transfected with miR-139-5p inhibitors or LMO4 siRNA for 48 h. (b) Cell viability was tested using a Cell Counting Kit-8 assay. (c, d) Cell apoptosis was assessed via flow cytometry. (e) ROS accumulation. (f) MDA contents. (g) Activities of SOD. (h) GSH-PX activities. (i) TNF-*α*, (j) IL-6, and (k) Cox-2 levels were measured using ELISA. A total of three independent experiments were carried out. Error bars represent the mean ± SD of ≥3 experiments. ^∗^*P* < 0.05. miR: microRNA; MDA: malondialdehyde; SOD: superoxide dismutase; GSH-PX: glutathione peroxidase; LMO4: LIM-only factor 4.

Then, ARPE-19 cells were transfected with miR-139-5p inhibitors or LMO4 siRNA. We confirmed that miR-139-5p was repressed by the inhibitors and LMO4 was reduced by the siRNA significantly (Figure 6(a)). Additionally, LMO4 siRNA largely reversed the effects of miR-139-5p inhibitors on cell proliferation and apoptosis *in vitro* (Figures 6(b)–6(d)). Knockdown of LMO4 also eliminated the effects of miR-139-5p on ROS accumulation (Figures 6(e)–6(h)) and decreased the effects of anti-miR-139-5p on the activation of inflammation (Figures 6(i)–6(k)). These findings indicated that miR-139-5p inhibited HG-induced cell damage by targeting LMO4.

## 4. Discussion

Recently, DR has been considered to be a serious vascular complication in diabetes, and multiple microRNAs have been recognized due to their biological role in DR progression [28–30]. For example, miR-15b is involved in DR in rats by regulating IRS-1 and Wnt/*β*-catenin signaling [31], while miR-29a serves a significant role in DR by targeting angiotensinogen in a DR rat model [32]. In addition, miR-9 can repress retinal neovascularization in DR rats by targeting VEGFA [33]. In the present study, it was demonstrated that miR-139-5p was significantly decreased by HG treatment, which suggested that miR-139-5p was involved in the DR development by targeting LMO4. We found that miR-139-5p overexpression increased ARPE-19 cell proliferation while reducing apoptosis of ARPE-19 cells. In addition, LMO4 served as a direct target of miR-139-5p as confirmed by a dual-luciferase reporter assay.

miR-139-5p serves an important role in diabetes. For instance, knockdown of miR-139-5p induces the functions of liraglutide on the diabetic rat pancreas by targeting IRS1 [34]. It has also been shown that the long noncoding RNA Gomafu can increase Foxo1 expression to enhance hepatic insulin resistance by regulating miR-139-5p [35]. Additionally, protein interacting with PRKCA 1 can attenuate HG-induced pancreatic *β* cell death by regulating PI3K/Akt, which can be modulated by miR-139-5p [36]. The regulatory mechanisms of miR-139-5p in diabetes are diverse. The present study identified that miR-139-5p expression was significantly downregulated in patients with DR, and it was decreased by HG in ARPE-19 cells in a time-dependent manner. Furthermore, overexpression of miR-139-5p repressed ARPE-19 cell damage by enhancing cell proliferation and inhibiting cell apoptosis, oxidative stress, and inflammation. Our study reported a significant role of miR-139-5p in DR development.

It has been reported that LMO4 can modulate TGF-*β* in epithelial cells by interacting with Smad [37]. LMO4 is also required to balance the hypothalamic insulin pathway [38]. In addition, LMO4 has been revealed to modulate 3T3-L1 preadipocyte proliferation and differentiation [39]. LMO4 is also able to inhibit protein tyrosine phosphatase 1B [40]. However, the role of LMO4 in DR remains poorly known. In the present study, LMO4 was identified to be a target of miR-139-5p. Moreover, it was found that LMO4 was induced by HG in ARPE-19 cells, and loss of LMO4 markedly reversed the effects of anti-miR-139-5p. However, the lack of in vivo experimentation is a great limitation in our study. In our future study, miR-139-5p and LMO4 would be modulated using lentivirus injection into the mice to confirm the in vitro results of our study. Collectively, we revealed the association between miR-139-5p and the LMO4 axis in DR. Furthermore, the detailed regulatory mechanism needs to be explored in the future experiments.

In conclusion, the present study demonstrated that miR-139-5p could protect ARPE-19 cell injury induced by HG via proproliferation, antiapoptosis, antioxidant activity, and anti-inflammation effects. These functions of miR-139-5p may be ascribed to targeting LMO4.

## Figures and Tables

**Table 1:** Characteristics of DR patients and the control group.

	Control (*n* = 30)	DR (*n* = 30)	*P* value
Age (years)	64.63 ± 2.95	71.6 ± 3.61	0.073
Gender (male/female)	17/13	19/11	0.692
Fasting blood glucose (mmol/l)	5.21 ± 0.35	8.53 ± 0.36	<0.001

**Table 2:** Primers used for real-time PCR.

Genes	Forward (5′-3′)	Reverse (5′-3′)
GAPDH	CAAGGTCATCCATGACAACTTTG	GTCCACCACCCTGTTGCTGTAG
LMO4	GGACAGTCGATTCCTGCGAG	TGTAGTGAAACCGATCTCCCG-
miR-139-5p	GCCTCTACAGTGCACGTGTCTC	CGCTGTTCTCATCTGTCTCGC
U6	ATTGGAACGATACAGAGAAGATT	GGAACGCTTCACGAATTTG

## Data Availability

The data used to support the findings of this study are included within the article.

## References

[1] Congdon N. G., Friedman D. S., Lietman T. (2003). Important causes of visual impairment in the world today. *Journal of the American Medical Association*.

[2] Durham J. T., Herman I. M. (2011). Microvascular modifications in diabetic retinopathy. *Current Diabetes Reports*.

[3] Das A., Stroud S., Mehta A., Rangasamy S. (2015). New treatments for diabetic retinopathy. *Diabetes, Obesity & Metabolism*.

[4] Kim D. I., Park M. J., Choi J. H., Lim S. K., Choi H. J., Park S. H. (2015). Hyperglycemia-induced GLP-1R downregulation causes RPE cell apoptosis. *The International Journal of Biochemistry & Cell Biology*.

[5] Malfait M., Gomez P., van Veen T. A. (2001). Effects of hyperglycemia and protein kinase C on connexin 43 expression in cultured rat retinal pigment epithelial cells. *The Journal of Membrane Biology*.

[6] Ponnalagu M., Subramani M., Jayadev C., Shetty R., Das D. (2017). Retinal pigment epithelium-secretome: a diabetic retinopathy perspective. *Cytokine*.

[7] Hayes J., Peruzzi P. P., Lawler S. (2014). MicroRNAs in cancer: biomarkers, functions and therapy. *Trends in molecular medicine*.

[8] Filipowicz W., Bhattacharyya S. N., Sonenberg N. (2008). Mechanisms of post-transcriptional regulation by microRNAs: are the answers in sight?. *Nature Reviews Genetics*.

[9] Mohr A. M., Mott J. L. (2015). Overview of microRNA biology. *Seminars in Liver Disease*.

[10] Hayes J., Peruzzi P. P., Lawler S. (2014). MicroRNAs in cancer: biomarkers, functions and therapy. *Trends in Molecular Medicine*.

[11] Rupaimoole R., Slack F. J. (2017). MicroRNA therapeutics: towards a new era for the management of cancer and other diseases. *Nature Reviews Drug Discovery*.

[12] Bartels C. L., Tsongalis G. J. (2009). MicroRNAs: novel biomarkers for human cancer. *Clinical Chemistry*.

[13] Kantharidis P., Wang B., Carew R. M., Lan H. Y. (2011). Diabetes complications: the microRNA perspective. *Diabetes*.

[14] Pandey A. K., Agarwal P., Kaur K., Datta M. (2009). MicroRNAs in diabetes: tiny players in big disease. *Cellular Physiology and Biochemistry: International Journal of Experimental Cellular Physiology, Biochemistry, and Pharmacology*.

[15] McArthur K., Feng B., Wu Y., Chen S., Chakrabarti S. (2011). MicroRNA-200b regulates vascular endothelial growth factor-mediated alterations in diabetic retinopathy. *Diabetes*.

[16] Natarajan R., Putta S., Kato M. (2012). MicroRNAs and diabetic complications. *Journal of Cardiovascular Translational Research*.

[17] Chen Q., Qiu F., Zhou K. (2017). Pathogenic role ofmicroRNA-21in diabetic retinopathy through downregulation of PPAR*α*. *Diabetes*.

[18] Fang S., Ma X., Guo S., Lu J. (2017). MicroRNA-126 inhibits cell viability and invasion in a diabetic retinopathy model via targeting IRS-1. *Oncology Letters*.

[19] Xia F., Sun J. J., Jiang Y. Q., Li C. F. (2018). MicroRNA-384-3p inhibits retinal neovascularization through targeting hexokinase 2 in mice with diabetic retinopathy. *Journal of Cellular Physiology*.

[20] Shi Y. K., Guo Y. H. (2018). miR-139-5p suppresses osteosarcoma cell growth and invasion through regulating DNMT1. *Biochemical and Biophysical Research Communications*.

[21] Li P., Xiao Z., Luo J., Zhang Y., Lin L. (2019). miR-139-5p, miR-940 and miR-193a-5p inhibit the growth of hepatocellular carcinoma by targeting SPOCK1. *Journal of Cellular and Molecular Medicine*.

[22] Wang K., Jin J., Ma T., Zhai H. (2017). miR-139-5p inhibits the tumorigenesis and progression of oral squamous carcinoma cells by targeting HOXA9. *Journal of Cellular and Molecular Medicine*.

[23] Hua S., Lei L., Deng L. (2018). miR-139-5p inhibits aerobic glycolysis, cell proliferation, migration, and invasion in hepatocellular carcinoma via a reciprocal regulatory interaction with ETS1. *Oncogene*.

[24] Zhang H., Sun Z., Yu L., Sun J. (2017). MiR-139-5p inhibits proliferation and promoted apoptosis of human airway smooth muscle cells by downregulating the Brg1 gene. *Respiratory Physiology & Neurobiology*.

[25] Papangeli I., Kim J., Maier I. (2016). MicroRNA 139-5p coordinates APLNR-CXCR4 crosstalk during vascular maturation. *Nature Communications*.

[26] Wu Y., Li H., Xie J., Wang F., Cao D., Lou Y. (2020). miR-139-5p affects cell proliferation, migration and adipogenesis by targeting insulin‑like growth factor 1 receptor in hemangioma stem cells. *International Journal of Molecular Medicine*.

[27] Walz J. M., Wecker T., Zhang P. P. (2019). Impact of angiogenic activation and inhibition on miRNA profiles of human retinal endothelial cells. *Experimental Eye Research*.

[28] Ting D. S., Tan K. A., Phua V., Tan G. S., Wong C. W., Wong T. Y. (2016). Biomarkers of diabetic retinopathy. *Current Diabetes Reports*.

[29] Gong Q., Su G. (2017). Roles of miRNAs and long noncoding RNAs in the progression of diabetic retinopathy. *Bioscience Reports*.

[30] Pelikanova T. (2016). Diabetic retinopathy: pathogenesis and therapeutic implications. *Vnitrni Lekarstvi*.

[31] Liu H. W., Meng Y., Ren Y. B., Sun P. (2018). MicroRNA-15b participates in diabetic retinopathy in rats through regulating IRS-1 via Wnt/*β*-catenin pathway. *European Review for Medical and Pharmacological Sciences*.

[32] Zhang L. Q., Cui H., Wang L., Fang X., Su S. (2017). Role of microRNA-29a in the development of diabetic retinopathy by targeting AGT gene in a rat model. *Experimental and Molecular Pathology*.

[33] Liu W. L. (2019). MicroRNA-9 inhibits retinal neovascularization in rats with diabetic retinopathy by targeting vascular endothelial growth factor A. *Journal of Cellular Biochemistry*.

[34] Li J., Su L., Gong Y. Y. (2017). Downregulation of miR-139-5p contributes to the antiapoptotic effect of liraglutide on the diabetic rat pancreas and INS-1 cells by targeting IRS1. *PloS One*.

[35] Yan C., Li J., Feng S., Li Y., Tan L. (2018). Long noncoding RNA Gomafu upregulates Foxo1 expression to promote hepatic insulin resistance by sponging miR-139-5p. *Cell Death & Disease*.

[36] Qiu H., Ma L., Feng F. (2020). PICK1 attenuates high glucose-induced pancreatic *β*-cell death through the PI3K/Akt pathway and is negatively regulated by miR-139-5p. *Biochemical and Biophysical Research Communications*.

[37] Lu Z., Lam K. S., Wang N., Xu X., Cortes M., Andersen B. (2006). LMO4 can interact with Smad proteins and modulate transforming growth factor- *β* signaling in epithelial cells. *Oncogene*.

[38] Pandey N. R., Zhou X., Zaman T. (2014). LMO4 is required to maintain hypothalamic insulin signaling. *Biochemical and Biophysical Research Communications*.

[39] Wang N., Wang X., Shi M. (2013). LMO4 modulates proliferation and differentiation of 3T3-L1 preadipocytes. *FEBS Letters*.

[40] Pandey N. R., Zhou X., Qin Z. (2013). The LIM domain only 4 protein is a metabolic responsive inhibitor of protein tyrosine phosphatase 1B that controls hypothalamic leptin signaling. *The Journal of Neuroscience: The Official Journal of the Society for Neuroscience*.

